# Two-minute disconnection technique with a double-lumen tube to speed the collapse of the non-ventilated lung for one-lung ventilation in thoracoscopic surgery

**DOI:** 10.1186/s12871-017-0371-x

**Published:** 2017-06-15

**Authors:** Qiongzhen Li, Xiaofeng Zhang, Jingxiang Wu, Meiying Xu

**Affiliations:** Department of Anesthesiology of Shanghai Chest Hospital, Shanghai Jiaotong University, Shanghai, 200030 China

**Keywords:** Disconnection ventilation, One-lung ventilation, Thoracoscopic surgery, Lung collapse, Cerebral tissue oxygen saturation

## Abstract

**Background:**

Thoracic surgery requires the effective collapse of the non-ventilated lung. In the majority of cases, we accomplished, accelerated lung collapse using a double-lumen tube (DLT). We hypothesized that using the two-minute disconnection technique with a DLT would improve lung collapse during subsequent one-lung ventilation.

**Methods:**

Fifty patients undergoing thoracoscopic surgery with physical classification I or II according to the American Society of Anesthesiologists were randomly divided into two groups for respiratory management of one-lung ventilation (OLV). In group N, OLV was initiated after the DLT was disconnected for 2 min; the initiation time began when the surgeon made the skin incision. In group C, OLV was initiated when the surgeon commenced the skin incision and scored the quality of lung collapse (using a four-point ordinal scale). The surgeon’s satisfaction or comfort with the surgical conditions was assessed using a visual analogue scale. rSO_2_ level, mean arterial pressure, pulse oxygen saturation, arterial blood gas analysis, intraoperative hypoxaemia, intraoperative use of CPAP during OLV, and awakening time were determined in patients at the following time points: while inhaling air (T_0_), after anaesthesia induction andinhaling 100% oxygen in the supine position under double lung ventilation for five mins (T_1_), at two mins after skin incision (T_2_), at ten mins after skin incision (T_3_), and after the lung recruitment manoeuvres and inhaling 50% oxygen for five mins (T_4_).

**Results:**

The two-minute disconnection technique was associated with a significantly shorter time to total lung collapse compared to that of the conventional OLV ventilation method (15 mins vs 22 mins, respectively; *P* < 0.001), and the overall surgeon’s satisfaction was higher (9 vs 7, respectively; *P* < 0.001). At T_2_, the PaCO_2_, left rSO_2_ and right rSO_2_ were higher in group N than in group C. There were no statistically significant differences between the incidence of intraoperative hypoxaemia and intraoperative use of CPAP during OLV (10% vs 5%, respectively; *P* = 1.000), duration of awakening (18 mins vs 19 mins, respectively; *P* = 0.616).

**Conclusions:**

A two-minute disconnection technique using a double-lumen tube was used to speed the collapse of the non-ventilated lung during one-lung ventilation for thoracoscopic surgery. The surgeon was satisfied with the surgical conditions.

**Trial registration:**

Chinese Clinical Trial Registry number, ChiCTR-IPR-17010352. Registered on Jan, 7, 2017.

## Background

Both right and left video-assisted thoracoscopic surgery (VATS) commonly require effective lung collapse of the non-ventilated lung to facilitate lung exposure. Most British and Middle Eastern thoracic anaesthesiologists are likely to use a double-lumen endobronchial tube (DLT) as their first choice for operative lung collapse [1, 2].

Lung collapse quality is a major concern when selecting DLT for one-lung ventilation (OLV), because it can permit adequate surgical exposure and promote insertion of the trocars. A previous study demonstrated that lung collapse was the distribution for two phases. The first, rapid phase of lung collapse occurs as soon as ambient air freely enters the thoracic cavity; the lungs will undergo rapid partial collapse because of the inherent elastic recoil that occurs within 1 min [3]. When the rapid collapse ceases, a consequence of small airway closure, the second, slow phase of lung collapse ensues; this is dependent on absorption atelectasis and continuous gaseous diffusion. However, prior to the thoracic cavity being opened to the atmosphere, with each breath of positive-pressure, ventilation to the ventilated lung generates pressure that is transmitted to the opposite hemithorax, resulting in a mean (range) tidal movement of 134 (65–265) ml of gas in the non-ventilated lung [4].

Using a disconnection technique during VATS for pneumothorax has been shown to have a comparable degree of lung collapse [5]. Different disconnection times have been described to accelerate lung collapse, with several studies demonstrating a time ranging from 15 to 60 s [5–7]. However, the proper time for disconnection and the monitoring technique were not provided.

The primary aim of this study was to confirm the efficacy of the two-minute disconnection technique with a double-lumen tube to speed the collapse of the non-ventilated lung during one-lung ventilation for thoracoscopic surgery and to evaluate the safety of the two-minute disconnection technique.

## Methods

### Study design and patient population

This randomized, prospective double-blind study enrolled 50 patients from Jan, 7, 2017 to Feb, 1, 2017. This study was approved by the Institutional Review Board of Shanghai Jiaotong University Shanghai Chest Hospital (KS-2015-32), and the patients provided written informed consent (Chinese Clinical Trial Registry number, ChiCTR-IPR-17010352).

### Criteria for inclusion and exclusion

The patients were classified as physical status I or II according to the American Society of Anesthesiologists, ranged in age between 20 and 65 years old and were scheduled to undergo video-assisted thoracoscopic surgery requiring OLV. The exclusion criteria included patients with the following conditions: abnormal expiratory recoil (FEV_1_ < 70% of predicted value), chronic obstructive pulmonary disease (COPD) or severe asthma, a history of thoracic surgery, a risk of blood or infected secretions contaminating the dependent lung or NewYork Heart Association (NYHA) heart failure class II to IV and cerebrovascular disease.

### Preoperative preparations and anaesthesia protocol

The patients received no premedication. When the patients arrived in the operating room, they were monitored by pulse oximetry, electrocardiography (ECG), non-invasive blood pressure (NIBP), bispectral index (BIS) values and bilateral frontal rSO_2_ (MNIR-100; Mingxi Medical, Chongqin, China). Using a catheter inserted into a peripheral vein, 6 ml/kg of crystalloid was injected into the patients. Invasive blood pressure monitoring was achieved by cannulating the radial artery after administering lidocaine local anaesthesia. The patients were randomly assigned to groups N and C. After anaesthesia was induced with a target-controlled infusion (TCI) of 2% propofol, at an effect-site concentration (Ce) of 4 μg/ml, 0.6 μg/kg sufentanil, 0.2 mg/kg cisatracurium, and 1 μg/kg dexmedetomidine (DEX) for 10 min, the patients were intubated with a non-operation lateral DLT (size F35 for women and size F37 for men) by experienced thoracic anaesthesiologists involved in the study, and the correct position was confirmed using a fibreoptic bronchoscope (FOB). In the initial two-lung ventilation (TLV) and one-lung ventilation (OLV) period, the tidal volume was 7 ml/kg, the respiratory rate was 12 bpm, and the I/E ratio was 1:2. Anaesthesia was maintained using 0.12 mg/kg/h cisatracurium and 2% propofol Ce at 2–3 μg/ml titrated to maintain BIS between 40 and 50; mean arterial blood pressures (MAPs) and heart rates (HRs) were 20% less than the baseline values. The patient was placed in a lateral decubitus position after right internal jugular central venous catheterization. The correct DLT position was confirmed again using a FOB. In group N, OLV was initiated after the DLT was disconnected for 2 min; the initiation time commenced when the surgeon performed the skin incision. In group C, OLV was initiated when the surgeon started to incise the skin. In both groups, anaesthesia induction and OLV were initiated and maintained using 100% oxygen. The VATS procedure enabled the surgeon to explore the pleura cavity using a 30° video thoracoscopic camera; through a 1.5 cm single skin incision and one to three trocars, the thoracoscopic instruments could move through the lung. After opening the pleura, when the lung was visible, the surgeon started to score the quality of lung collapse using a four-point ordinal scale. Campos et al. and Mourisse et al. [8, 9]^,^ 1 = extremely poor-no collapse of lung; 2 = poor-partial collapse of lung with interference with surgical exposure; 3 = good-total collapse, but the lung still had some residual air; and 4 = excellent-complete collapse of lung with perfect surgical exposure). At the end of the surgery, after the lung recruitment manoeuvre, the inspiratory pressure increased to 40 cmH_2_O for 10 s. Two-lung ventilation was maintained with 50% oxygen and 5 cmH_2_O of positive end expiratory pressure (PEEP). When the operation was completed, the patients were discharged to the PACU.

### Measurements

MBP, HR, rSO_2_ levels, SpO_2_ values, and blood gas analysis outcomes were recorded at the following time points: when inhaling air (T_0_), after inhaling 100% oxygen while in the supine position and under double lung ventilation for five mins (T_1_), at two mins after the skin incision (T_2_), at 10 min after the skin incision (T_3_) and after the lung recruitment manoeuvers and inhaling 50% oxygen for five mins (T_4_).

The primary outcome was the time needed for complete lung collapse between the two groups. The outcome was measured from the start of the skin incision to the time of total lung collapse and was graded between 3 to 4 according to the video viewed by a surgeon who was blinded to the lung collapse technique. The secondary outcomes were the surgeon’s satisfaction with the surgical conditions using a visual analogue scale (0 = unsatisfied; 10 = very satisfied), the development of hypoxaemia (SpO_2_ < 90%) and the need for CPAP during OLV. The same surgeons who were blinded to the lung collapse technique performed all surgical procedures. All of the data were recorded by another anaesthesiologist who was unfamiliar with the lung collapse technique.

### Statistical analysis

The previous study evaluated the degree of lung collapse in which the number of patients per group was 16 and detected global differences among the three groups [6]. The number of patients in each group was determined by a power calculation based on the results of Campos et al. [8] It was calculated that 18 patients were needed per group assuming an α risk of 0.05, a β risk of 0.10 and a mean difference of 50%. Because of the risk of failure with regard to lung adhesion or intubation, we decided to enrol 50 patients in the trial. The data are expressed as the mean ± standard deviation (SD) or as the number of patients. The statistical analyses were performed using a paired Student’s t-test. The categorical variables were performed using the χ^2^-test. We performed the statistical analyses using SPSS version 21.0 (SPSS Inc. Chicago, IL, USA). A *p-*value < 0.05 was considered statistically significant.

## Results

**Fig. 1:**
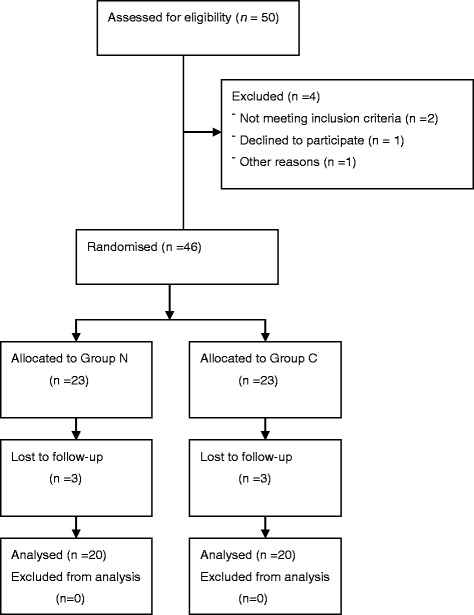
CONSORT flow diagram

A total of 50 patients were enrolled in this study; however, five patients in each group were excluded from the data analysis. The final analyses included 20 patients per group (Fig. 1).

Patients’ characteristics were not different between the two groups (Table 1).

**Table 1:** Patient characteristics

Variable	Group N (*n* = 20)	Group C (*n* = 20)
Age (years)	44 ± 9.8	46 ± 12.8
Sex (M/F)	13/7	14/6
Weight (kg)	62.2 ± 13.3	64.2 ± 12.4
Height (cm)	164 ± 9.0	166 ± 8.0
ASA class I/II	5/15	4/16
FEV_1_ (% of the predicted value)	78.1 ± 4.5	76.4 ± 4.2
FVC (% of the predicted value)	76.4 ± 4.2	74.8 ± 2.9
Side of thoracotomy (right/left) (n)	11/9	12/8
Type of surgery (n)		
Wedge resection	5	7
Segmentectomy	9	8
Lobectomy	6	5

The data are expressed as the mean ± SD values or the number of patients. ASA, American Society of Anesthesiologists

The time required for lung collapse was significantly shorter in group N than in group C (15 ± 3.7 mins vs 22 ± 3.6 mins) during one-lung ventilation. Additionally, the overall surgeon’s satisfaction was higher in group N than in group C (9 ± 0.6 vs 7 ± 1.2). Intraoperative hypoxaemia and the use of CPAP during OLV did not significantly differ between the two groups. The duration of anaesthesia and awakening was not significantly different between the two groups (Table 2).

**Table 2:** Clinical outcomes

Variable	Group N (*n* = 20)	Group C (*n* = 20)	*P*
Time required for lung collapse (min)	15 ± 3.7	22 ± 3.6	<0.001
Time required for pleural opening (s)	85 ± 6.7	88 ± 6.6	=0.176
Overall satisfaction of the surgeon	9 ± 0.6	7 ± 1.2	<0.001
Intraoperative hypoxaemia (n)	2	1	=1.000
Intraoperative use of CPAP during OLV (n)	2	1	=1.000
Duration of anaesthesia (min)	124 ± 27.9	125 ± 28.9	=0.847
Duration of awakening (min)	18 ± 3.2	19 ± 5.3	=0.616

The data are expressed as the mean ± SD values or the number of patients. CPAP, continuous positive airway pressure; OLV, one-lung ventilation. *P* < 0.05 was statistically significant compared with group C

At T_2_, the PaCO_2_ levels were higher in group N than in group C (47.7 ± 2.9 vs 39.2 ± 3.4); however, the PaO_2_ levels were lower in group N than in group C (234 ± 81.1 vs 335 ± 33.4) (Table 3).

**Table 3:** Blood gas analysis

Variable	Group	T_0_	T_1_	T_2_	T_3_	T_4_
pH	N (*n* = 20)	7.39 ± 0.03	7.40 ± 0.04	7.34 ± 0.04*	7.37 ± 0.03*	7.36 ± 0.04*
	C (*n* = 20)	7.37 ± 0.03	7.38 ± 0.03	7.35 ± 0.04	7.35 ± 0.03*	7.36 ± 0.04
PaCO_2_	N (*n* = 20)	36.1 ± 2.0	37.1 ± 3.1	47.7 ± 2.9*†	44.1 ± 4.5*	38.5 ± 4.2 *
(mmHg)	C (*n* = 20)	36.9 ± 1.8	37.0 ± 3.9	39.2 ± 3.4*	43.6 ± 2.3*	39.3 ± 4.9*
PaO_2_	N (*n* = 20)	82 ± 3.1	418 ± 48.9*	234 ± 81.1*†	239 ± 77.7*	272 ± 94.3*
(mmHg)	C (*n* = 20)	84 ± 3.5	428 ± 47.9*	335 ± 33.4*	230 ± 70.9*	282 ± 96.0*

The data are expressed as the mean ± SD values. * *P* < 0.05 compared with T_0_. ^†^ *P* < 0.05 compared with group C

The MBP and HR were not significantly different at any time point between the two groups. At T_2_, the left rSO_2_ and right rSO_2_ were higher in group N than in group C (Table 4).

**Table 4:** MAP, HR, SpO_2_ and rSO_2_ at different time points

Variable	Group	T_0_	T_1_	T_2_	T_3_	T_4_
MAP	N (*n* = 20)	76 ± 11.1	73 ± 11.3	72 ± 6.1	74 ± 10.3	71 ± 7.8
(mmHg)	C (*n* = 20)	74 ± 9.2	72 ± 7.8	73 ± 7.7	72 ± 9.3	72 ± 7.0
HR	N (*n* = 20)	71 ± 11.1	63 ± 6.6*	68 ± 7.5	69 ± 6.1	70 ± 8.0
(bpm)	C (*n* = 20)	73 ± 11.4	65 ± 7.8*	67 ± 6.9	70 ± 6.9	69 ± 7.3
SpO_2_	N (*n* = 20)	96 ± 0.7	99 ± 0.8*	99 ± 2.3*	99 ± 1.2*	99 ± 1.0*
(%)	C (*n* = 20)	95 ± 0.9	99 ± 0.9*	99 ± 1.3*	99 ± 1.1*	99 ± 0.9*
Left rSO_2_	N (*n* = 20)	69 ± 4.7	75 ± 4.1*	78 ± 3.4*†	70 ± 7.7	71 ± 4.1
(%)	C (*n* = 20)	70 ± 4.5	75 ± 5.5*	74 ± 5.9*	71 ± 6.9	70 ± 4.4
Right rSO_2_	N (*n* = 20)	68 ± 4.3	74 ± 5.1*	77 ± 4.3*†	71 ± 4.9	70 ± 4.1
(%)	C (*n* = 20)	69 ± 4.8	75 ± 5.0*	74 ± 5.2*	72 ± 4.9*	71 ± 3.6

The data are expressed as the mean ± SD values. * *P* < 0.05 compared with T_0_. ^†^ *P* < 0.05 compared with group C

## Discussion

This study found that, compared with conventional one-lung ventilation, the two-minute disconnection technique before one-lung ventilation was associated with a shorter time to achieve complete lung collapse (15 mins vs 22 mins). This outcome is in accordance with the study by Young [5], in which the disconnection technique was associated with a shorter time to lung collapse. Campos [8] showed that DLT took 17:54 min:s to achieve lung collapse. Several methods have been described to accelerate lung collapse, including denitrogenation of the lung with an FiO_2_ of 1.0 [10], filling the lung with 50% nitrous oxide [10, 11], and using disconnection [5, 6, 12] and suction techniques [8].

This study also found that the two-minute disconnection technique before one-lung ventilation did not cause hypoxaemia, and that the rSO_2_ increased two mins after the skin incision. This occurred because PaCO_2_ levels increased or decreased by 1 mmHg, and the cerebral blood flow increased or decreased by 2 ml/100 g·min [13]. Tisdall found that lower ventilation can cause hypercapnia, leading to cerebrovascular expansion by increasing the cerebral blood flow to increase the brain tissue oxygen and index [14]. Continuously monitoring rSO_2_ during the perioperative period can considerably reduce the risk of cerebral ischaemia and hypoxia [15].

Compared with conventional one-lung ventilation, the two-minute disconnection technique before one-lung ventilation can accelerate the collapse of the non-ventilated lung and improve the overall surgeon’s satisfaction. Although there was a permissive hypercapnia during the operation, hypercapnia was corrected over time; furthermore, the rSO_2_ levels did not decrease. There was no significant difference in the patients’ intraoperative hypoxaemia or in the duration of awakening.

Many surgical procedures are required for OLV, such as lung surgery, oesophageal surgery, and minimally invasive-cardiac surgery. All of these surgical procedures require excellent lung collapse for optimal surgical exposure without having to compress the lung parenchyma facilitate the surgeon’s dissection and reduce both the operating time and incidence of postoperative complications [16]. The definition of lung collapse time changes from one study to another, from the opening of the pleura [8] or from the start of one-lung ventilation [17]. We calculated lung collapse time from the start of the skin incision, and both groups demonstrated a similar time to perform the pleural opening. We chose the two-minute disconnection technique because it was considered to be effective during both the first phase of lung collapse that occurs within 1 min [3] and the time from skin incision to pleura opening.

There are several limitations in the present study. First, we assessed lung collapse using the surgeon’s rating scale; this method was less objective. Second, we did not aim to test the incidence of contamination with secretions or blood in either lung. Third, we excluded geriatric patients (>65 years of age). Fourth, we did not define postoperative haemodynamic and respiratory conditions and complications. Therefore, additional studies are warranted to investigate the efficacy and safety of the two-minute disconnection technique with a double-lumen tube to speed the collapse of the non-ventilated lung for one-lung ventilation during thoracoscopic surgery in these patients.

## Conclusions

The use of the two-minute disconnection technique using a double-lumen tube offers an effective method to accelerate the collapse of the non-ventilated lung during one-lung ventilation for thoracoscopic surgery. This simple technique could facilitate thoracic surgery without causing hypoxia.

## Abbreviations

ASA: American Society of Anesthesiologists
BIS: Bispectral index
Ce: Effect-site concentration
COPD: Chronic obstructive pulmonary disease
CPAP: Continuous positive airway pressure
DEX: Dexmedetomidine
DLT: Double-lumen tube
ECG: Electrocardiography
FOB: Fibreoptic bronchoscopy
HR: Heart rate
I:E: Inspiration/expiration
MBP: Mean blood pressure
NIBP: Non-invasive blood pressure
NYHA: NewYork Heart Association
OLV: One-lung ventilation
PACU: Post-anaesthetic care unit
PEEP: Positive end expiratory pressure
rSO_2_: Regional cerebral oxygen saturation
SD: Standard deviation
SpO_2_: Pulse oxygen saturation
TCI: Target-controlled infusion
TLV: Two-lung ventilation
VATS: Video-assisted thoracoscopic surgery
